# Supplementary material for: Anomalous formation of trihydrogen cations from water on nanoparticles
Source: Nat Commun. 2021 Jun 22;12:3839. doi: 10.1038/s41467-021-24175-9 (PMC8219811; doi:10.1038/s41467-021-24175-9)
Supplement: Supplementary file 1 — Supplementary Information [file 41467_2021_24175_MOESM1_ESM.pdf]

Supplementary Information to Anomalous Formation of Trihydrogen Cations from Water on Nanoparticles

By M. Said Alghabra *et al.*

Supplementary Method 1- Nanoparticle preparation and characterization:

The silica nanoparticles obtained from nanoComposix were fabricated via the condensation of silanes to form nanoparticles composed of an amorphous network of silicon and oxygen using the Stöber method[1]. The polydispersity of the particles was about 2.8% for the 100 nm and 5.9% for the 300 nm particles. The nanoparticles were washed extensively in deionized water and dried to ensure that they are exclusively covered by silanol and that they are clean of any residual reactants. For analysis of chemical moieties adsorbed on the surface of the nanoparticles, FTIR spectra were recorded. For this, we used a JASCO FT/IR- 6300 series spectrometer attached to a multiple reflection ATR accessory equipped with diamond crystal optics. Each curve represents 20 scans in the range from 400 to 4000 cm^{-1} with 2 cm^{-1} resolution. The FTIR spectra are shown in Figure S3.

.

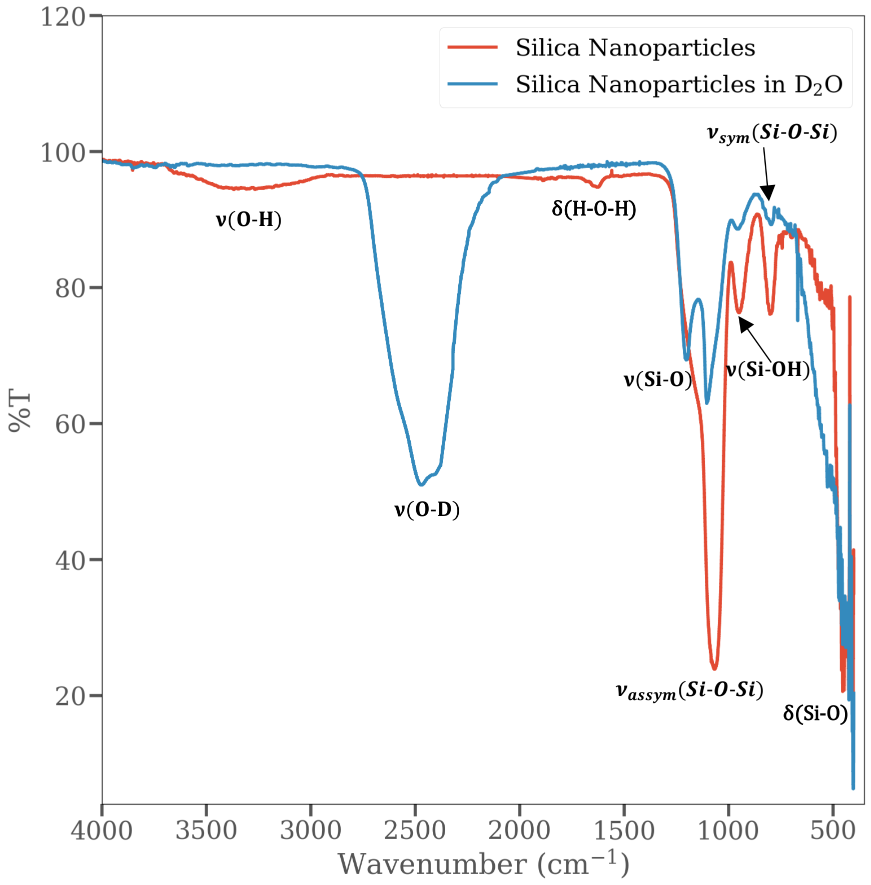

Supplementary Figure 1 ***ATR-FTIR transmittance spectra****. Spectra for 100nm SiO_2 powder nanoparticles and the same nanoparticles suspended in D_2O. While those spectra show the different stretching and bending bands for SiOH, SiOSi, SiO and OD, none of the spectra shows any signature of the IR modes in the regions 2800-3000 and 1100-1750 cm^{-1}, which could be associated to any organic attachments to the surface of the nanoparticles.*

.

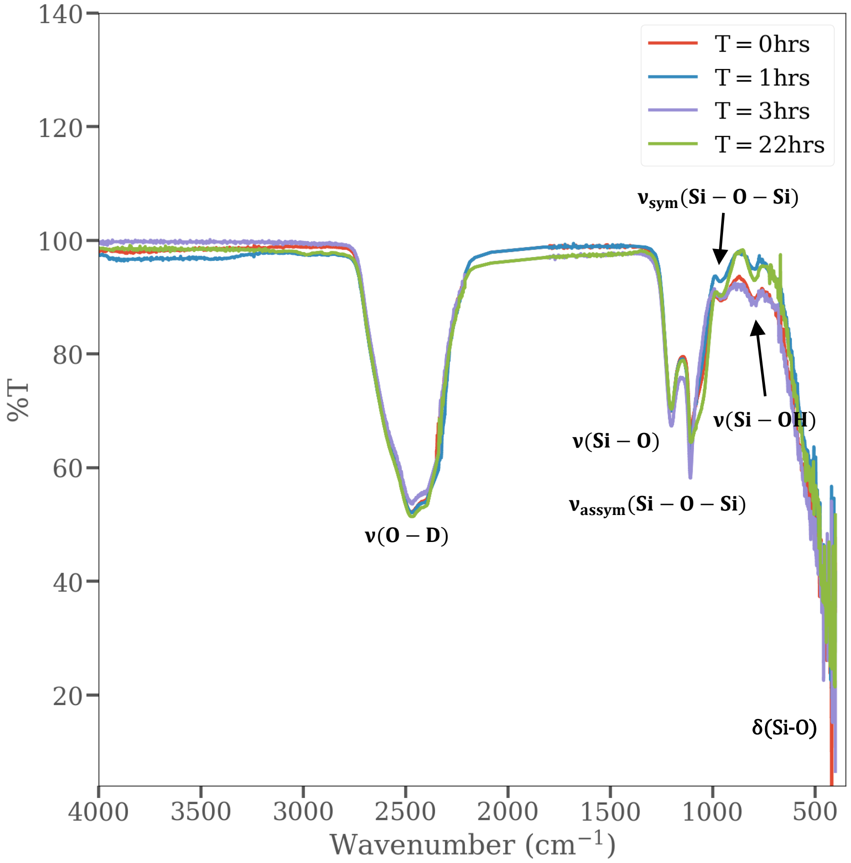

Supplementary Figure 2 ***ATR-FTIR transmittance spectra overtime.*** *Spectra of the silica nanoparticle immersed in D_2O and collected at different time intervals from the moment the particles were immersed (T= 0 hrs) until 22 hours (T = 22 hrs) after immersion. The spectra did not exhibit any change overtime and showed no indication of detectable H-D exchange between D_2O and SiOH, even over the course of 22 hrs.*

Supplementary Method 2-Time of flight (ToF) spectra obtained for nanoparticles and background gases:

Comparison between time of flight spectra for ions emitted from the surfaces of 100 nm silica nanoparticles inhabited by D_2O molecules and D_2O in the gas-phase, representing the background in the experiment with nanoparticles, as shown in Figure S4. Besides, the ionic fragments, the molecular ions are shown. Figure S5 displays the time of flight (TOF) spectra starting from H^+ ions up until the parent water ions demonstrating the absence of hydrocarbons from the used nanoparticles.

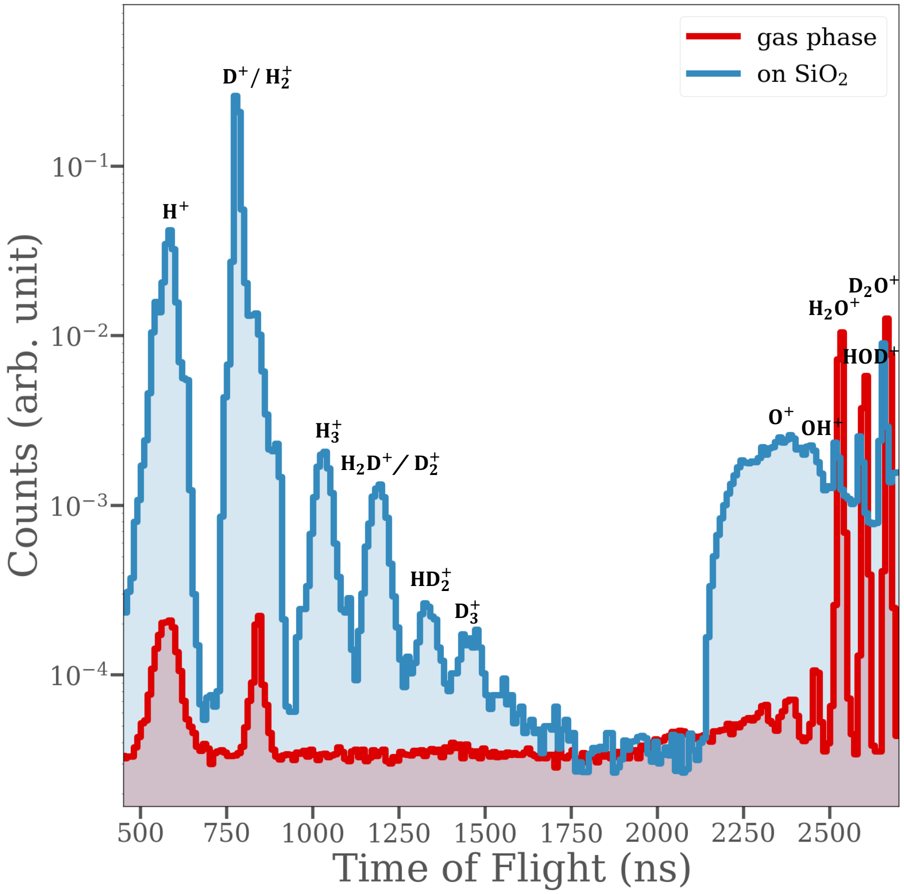

Supplementary Figure 3 ***Extended spectra for Ion emission from D_2O in gas-phase and on nanoparticles.*** *A comparison between ions emitted from the surfaces of 100nm silica nanoparticles inhabited by D_2O molecules at 3g/L concentration and D_2O in the gas phase representing the background of the experiment. Both experiments were irradiated by a laser intensity of $2 \times 10^{14}\ W/cm^2$. The comparison shows how H_3^+, D_3^+, and HD_2^+ can only be produced in the presence of nanoparticles.*

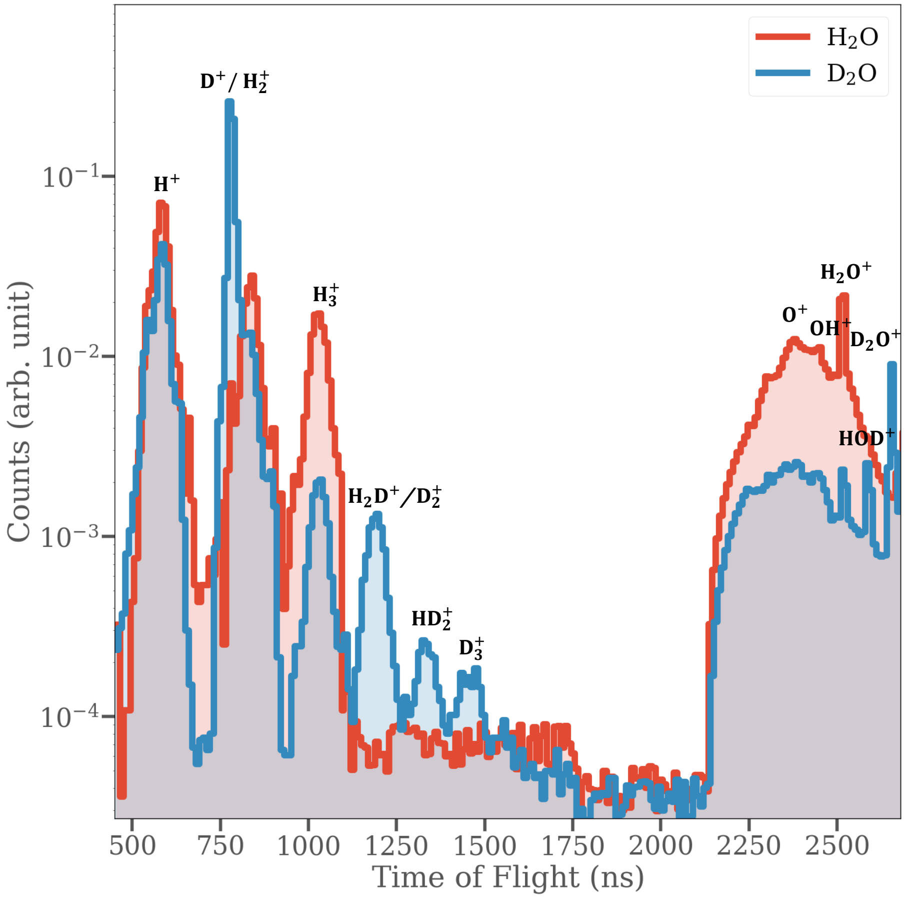

Supplementary Figure 4 ***Extended spectra for emitted ions from the surfaces of nanoparticles*** *A comparison between ions emitted from the surfaces of 100nm silica nanoparticles inhabited by H_2O and D_2O molecules at 3g/L concentration and irradiated by a laser intensity of $2 \times 10^{14}\ W/cm^2$. The presence of D_3^+ and HD_2^+ peaks in the ToF spectrum of D_2O adsorbed on nanoparticles presents unequivocal evidence about the source of the tri-deuterium ions. The spectra also demonstrate the absence of methyl groups and other hydrocarbons from the surfaces of used nanoparticles.*

Supplementary Method 3- Momentum and energy distributions

The section displays energy plots and momentum images of H_3^+ created on the surfaces of 100 nm and 300 nm nanoparticles, as well as D_3^+ generated on the surfaces of 100 nm nanoparticles. The figures highlight the similarities in angular and energy distribution for both H_3^+ and D_3^+ regardless of the nanoparticle size.

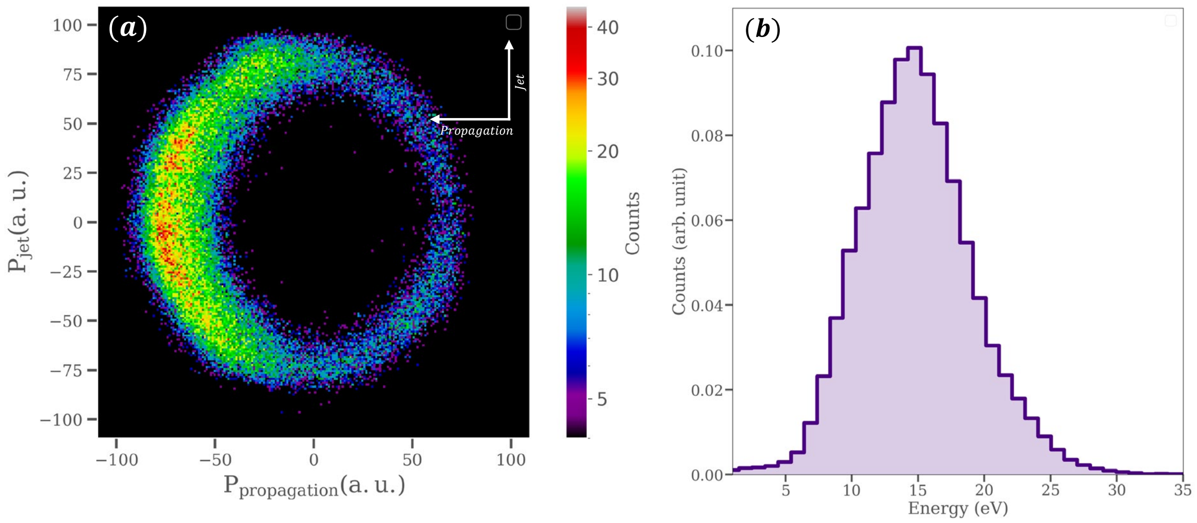

Supplementary Figure 5 ***Momentum and Energy of H_3^+ from 300nm SiO2 Particles.*** *A 2-D momentum image demonstrating the angular distribution in the laser propagation vs nanoparticles jet plane (cf. figure S1 for details about axes) where the laser polarization is perpendicular to this plane (a) and energy distribution (b) of H_3^+ emitted from the surface of 300 nm silica nanoparticles inhabited by H_2O molecules at a laser intensity of $2 \times 10^{14}\ W/cm^2$.*

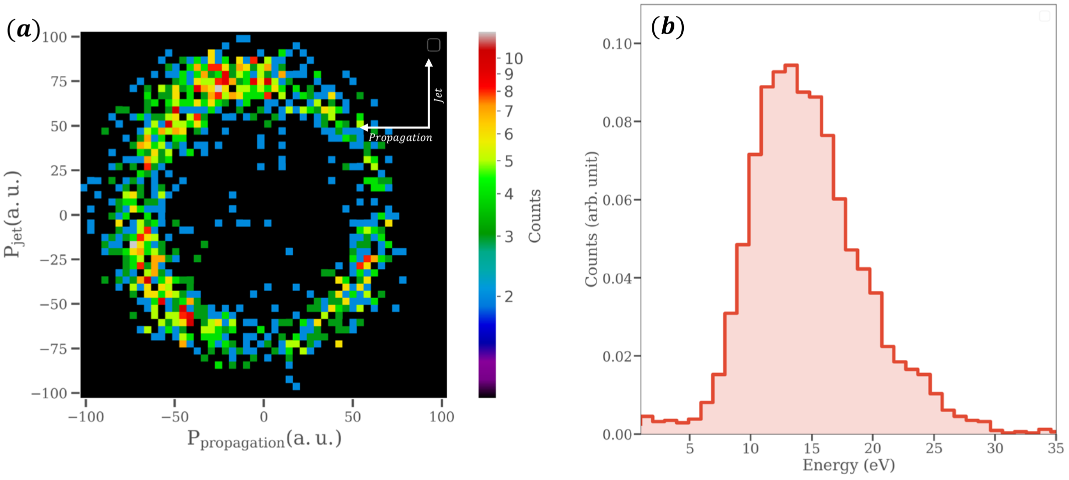

Supplementary Figure 6 ***Momentum and Energy of H_3^+ from 100nm SiO2 Particles.*** *A 2-D momentum image demonstrating the angular distribution in the laser propagation vs nanoparticles jet plane (cf. figure S1 for details about axes) where the laser polarization is perpendicular to this plane (a) and energy distribution (b) of H_3^+ emitted from the surface of 100nm silica nanoparticles inhabited by H_2O molecules at a laser intensity of $2 \times 10^{14}\ W/cm^2$.*

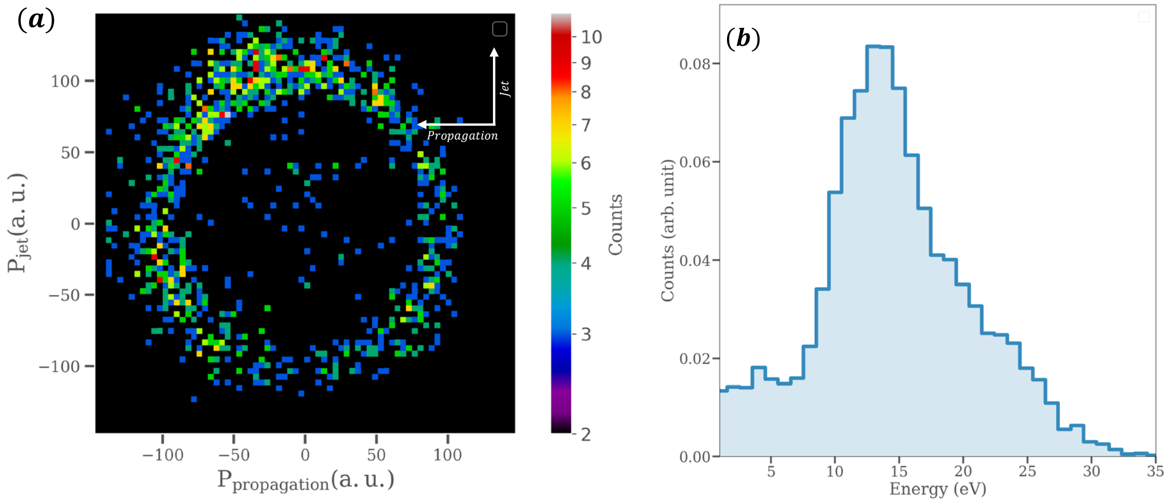

Supplementary Figure 7 ***Momentum and Energy of D_3^+ from 100nm SiO2 Particles.*** *A 2-D momentum image demonstrating the angular distribution in the laser propagation vs nanoparticles jet plane (cf. figure S1 for details about axes) where the laser polarization is perpendicular to this plane (a) and energy distribution (b) of D_3^+ emitted from the surface of 100 nm silica nanoparticles inhabited by D_2O molecules at a laser intensity of $2 \times 10^{14}\ W/cm^2$.*

Supplemementary References

1 Stöber, W., Fink, A. & Bohn, E. Controlled growth of monodisperse silica spheres in the micron size range. *Journal of Colloid and Interface Science* **26**, 62-69 (1968).
